# “It Can Hurt Your Heart”: A Co-Designed Cross-Sectional Survey Exploring Pacific People’s Understanding of Rheumatic Fever in Auckland, New Zealand

**DOI:** 10.3390/healthcare13222924

**Published:** 2025-11-15

**Authors:** Siobhan Tu’akoi, Malakai ‘Ofanoa, Samuela ‘Ofanoa, Melenaite Tohi, Maryann Heather, Hinamaha Lutui, Rose Lamont, Elizabeth Fanueli, Felicity Goodyear-Smith

**Affiliations:** 1 Pacific Health Department, School of Population Health, University of Auckland, Auckland 1023, New Zealand; 2 Etu Pasifika, Auckland 2104, New Zealand; 3 Southpoint Family Doctors, Auckland 2104, New Zealand; 4 Te Huinga Raukura ki Manurewa, South Auckland 2102, New Zealand; 5 Department of Social and Community Health, School of Population Health, University of Auckland, Auckland 1023, New Zealand; 6 Department of General Practice and Primary Health Care, University of Auckland, Auckland 1023, New Zealand

**Keywords:** rheumatic fever, Pacific, awareness, education, co-design, cross-sectional, survey, social media

## Abstract

**Background/Objectives**: Rheumatic fever is preventable and can be treated successfully; however, a lack of understanding of the disease and barriers to timely healthcare can impact outcomes. Pacific people in Aotearoa New Zealand experience inequitable burdens, and a Pacific community group and health professional network are working together to co-design education initiatives. This descriptive, mixed-methods study aimed to (1) explore Pacific people’s awareness and understanding of rheumatic fever, (2) describe where Pacific people access health information. **Methods**: An online survey co-developed with Pacific community members was run from December 2024 to February 2025. Questions related to sore throats, rheumatic fever, medication adherence, long-term outcomes and where Pacific people access health information. Quantitative data was analyzed descriptively using SPSS version 28 and open-ended qualitative responses were analyzed using an inductive content analysis approach. **Results**: A total of 400 Pacific respondents were included: 34% were aged 16–24 years and 66% were female. Based on the analysis, 71% of Pacific participants knew that a sore throat should always be checked by a health professional and 65.3% had heard of rheumatic fever. Fever and sore throats were commonly identified as symptoms of rheumatic fever, with joint pain, body aches and chest pain mentioned less. Barriers to health services such as cost, long waiting times and cultural factors were discussed as reasons why many Pacific people often utilize social media and online forums for health information. **Conclusions**: This study highlights gaps in rheumatic fever knowledge and thus opportunities for health education initiatives for Pacific communities, potentially utilizing social media and online platforms.

## 1. Introduction

Rheumatic fever is a global disease, with approximately 470,000 new cases every year [1]. It is an autoimmune response caused by an untreated Group A streptococcal infection, often presenting as a sore throat or skin infection, and is clinically diagnosed using the Jones criteria [1]. Recurrent episodes of rheumatic fever can damage heart valves and lead to rheumatic heart disease, the most common heart disease for people aged under 25 [2]. Risk factors for developing rheumatic fever are multifaceted and include household crowding, a family history of the disease and a lack of access to timely primary healthcare [3]. Initial streptococcal infections are typically treated with antibiotics, while intramuscular benzathine penicillin can effectively reduce recurrences of rheumatic fever and related heart disease [2]. Despite being preventable and in most cases treatable, barriers continue to exist that prevent people from receiving timely treatment both for streptococcal infections and for rheumatic fever.

Building public understanding and awareness about rheumatic fever is an important factor in enabling the early detection of symptoms and better primary and secondary prevention [4,5]. Globally, low levels of population awareness of rheumatic fever and rheumatic heart disease are reported [4,6,7]. A hospital-based study by Nkoke and colleagues in Cameroon, for example, interviewed 256 people being seen at an outpatient department (mean age of 34.4 years) [8]. The findings showed that 83% of participants had not heard of rheumatic heart disease and 70.3% did not know of any associations between a sore throat and rheumatic heart disease [8]. In Iran, Haghaninejad and colleagues aimed to evaluate the effectiveness of an educational intervention regarding the prevention of rheumatic fever [4]. Among 100 mothers of reproductive age, the findings showed increased mean scores of attitudes, practices and education related to rheumatic fever post intervention [4]. The authors recommended that community-based approaches to health promotion and education are needed in settings with high rates in order to improve the awareness and knowledge of rheumatic fever [4]. Ensuring the early identification of symptoms and better opportunities for effective, timely treatment is critical to preventing serious consequences.

In Aotearoa New Zealand (NZ), first episode rheumatic fever hospitalizations are increasing and currently sit at a national rate of 3.74 per 100,000 [9]. Pacific peoples in NZ experience first episode hospitalizations at a rate of 30.56 per 100,000 [9]. While ensuring communities are empowered to access knowledge regarding rheumatic fever prevention is one focus of NZ’s Rheumatic Fever Roadmap 2023–2028 [10], there is limited up-to-date research on the current levels of population awareness. Previous research primarily relates to a government-funded “Rheumatic Fever Prevention Programme” involving mass media campaigns between 2011 and 2017 that, despite reported improvements in awareness, was later critiqued for the stigma and blame it placed on the targeted communities [11,12]. Current data specifically focused on Pacific populations who experience the most significant rheumatic fever inequities is also lacking. One small-scale qualitative study in 2016 (*n* = 10) on Pacific people found a limited awareness and understanding overall and, in particular, of the prevention and the causal pathways to rheumatic fever [13]. To support the development of community-led health promotion strategies that might increase understanding [13], high-quality and up-to-date research is required.

A co-designed Pacific Research Collective in Auckland, NZ, is working towards Pacific-led initiatives that can support community understanding and awareness [14]. A Pacific community group, named the Pacific People’s Health Advisory Group (PPHAG), and a health professional network, called the Pacific Practice-based Research Network (PPBRN), are working together with university researchers to explore health issues and co-develop interventions. After PPHAG and PPBRN identified rheumatic fever as a key priority for Pacific populations in NZ, co-design workshops were undertaken to assess their views on existing interventions and to explore opportunities for innovative, Pacific-led approaches [14]. One key area the groups emphasized was the continued lack of understanding of rheumatic fever and the need for strengths-based, Pacific-led messaging [15]. To inform such strategies, the Pacific Research Collective decided that there was first a need to explore the current awareness of rheumatic fever in Pacific communities and understand how best health information might be translated for widespread reach. The Pacific population in Auckland was chosen due to them experiencing the highest rates of rheumatic fever in NZ. Additionally, although Pacific people are a diverse group with ancestry to Pacific Islands across Polynesia, Melanesia and Micronesia, there are common cultural values and traditions that unite them. This descriptive study therefore aimed to conduct a mixed-methods, cross-sectional survey of Pacific people aged 18 years and older living in Auckland, NZ. The study had the following two objectives:

1. To explore Pacific people’s awareness and understanding of rheumatic fever.
2. To describe where Pacific people usually access health-related information.

## 2. Materials and Methods

Ethical approval was obtained from the Auckland Health Research Ethics Committee, reference number AH27323, on 29 April 2024 for three years. The Strengthening the Reporting of Observational Studies in Epidemiology (STROBE) statement guidelines were followed for reporting (Supplementary Material S1).

### 2.1. Study Design and Participants

This cross-sectional study utilized an online, mixed-methods survey hosted on the Qualtrics platform. Participants were provided with a summary of the research on the main survey page, along with a linked participant information sheet describing the study in more detail. To consent to participate, respondents had to click “Yes” to the question “Do you consent to participate in this survey”. The inclusion criteria to participate was to (1) identify with at least one Pacific ethnicity, (2) be aged at least 16 years or older, (3) be currently living in Auckland, NZ, (4) be able to read and understand English, Tongan, Samoan, Cook Islands Māori, Niuean, Fijian or Tuvaluan. Respondents who did not provide consent, did not identify with at least one Pacific ethnicity, were younger than 16 years or who did not live in Auckland were excluded from the study. The digital sample size calculator Raosoft^®^ was utilized to estimate the sample size for this study (Raosoft, Inc., Seattle, WA, USA). Based on a population of 1,000,000, a confidence level of 95% and a 5% margin of error, it was estimated that approximately 384 responses would be required to accurately represent the Auckland Pacific population and provide reliable results.

Convenience sampling was used to recruit participants, firstly focused on networks of the Pacific research team, including different workplaces, sports clubs, Pacific churches and schools. Participants were also recruited through the community and professional networks of PPHAG and PPBRN. To ensure demographic representation of participants and reduce potential bias where possible, respondent characteristics were monitored throughout the period of data collection. This allowed for more targeted recruitment if certain groups had low response rates and enabled broad inclusion of education levels, age groups and ethnicities. Snowballing sampling approaches were additionally undertaken, with survey respondents encouraged to forward the survey onto people who may be eligible to participate. Recruitment occurred over a three-month period between December 2024 and February 2025.

### 2.2. Questionnaire Development

As part of a broader co-design project, the PPHAG community group and PPBRN health professional network worked with university researchers to develop and refine the questionnaire administered in this study. Based on previous input and discussion by the community groups and existing rheumatic fever survey tools, an initial set of survey questions was drafted. The content validity of questions was assessed by experts in the research team to ensure clarity and relevance of the questionnaire items for reflecting the intended concepts. The drafted survey questions were then brought to a further co-design workshop with the Pacific groups to review and refine them. Content equivalence was tested, ensuring that the questions could be understood and interpreted across different cultural groups [16]. Technical equivalence was reviewed to ensure that the format and mode of administration functioned as expected [16]. The community groups suggested changes to ensure readability and engagement, including restructuring the survey format, simplifying the delivery of questions, adding additional topics of interest and including images to make the survey more visually appealing in a Pacific way. After finalizing the content and format, survey questions were tested for readability via the Flesch–Kincaid test, resulting in a grade level of 5.98 (age range 11–12 years) or easy reading difficulty [17]. The survey was translated into Tongan, Samoan, Cook Islands Māori, Niuean, Fijian and Tuvaluan, representing the largest Pacific ethnicities in NZ. The final survey was piloted with a small group of Pacific people to ensure reliability and internal consistency before data collection.

### 2.3. Survey Measures

The final survey comprised three key sections of questions: (1) awareness and understanding of rheumatic fever, (2) where respondents usually obtain health-related information, (3) demographic information. In Section 1, ten questions explored the understanding of rheumatic fever, using a variety of multiple-choice and open-ended questions. Three questions were focused on sore throats in general, before a question asking, “Have you heard of rheumatic fever?”. The survey logic filtered participants who selected “no” to Section 2. Participants who selected “yes” then answered six further questions on rheumatic fever definitions, medication adherence and long-term outcomes. In Section 2, two questions were asked regarding where respondents usually obtain health-related information, firstly for themselves, and secondly, their view on where Pacific people, in general, obtain health information. Section 3 was focused on demographic data, including gender, age range, education level and ethnicity. Ethnicity was collected using a total response categorization method, whereby respondents can select as many ethnic groups as they choose to identify with. While this meant that ethnic group counts would be greater than the total number of respondents, it ensured that the full diversity of respondents was represented and that multiple ethnic groups could be reported [18]. The full survey, including questions and response options, can be found in Supplementary Material S2.

### 2.4. Statistical Analysis

Quantitative data were entered and cleaned in Microsoft Excel, before being imported into IBM SPSS Statistics 28 for analysis. Responses that were ineligible or unconsented were removed. Due to the small proportion of missing data, complete case analysis was applied to include only cases with no missing values [19]. Questions that permitted participants to select multiple categories at once had response options coded as separate binary variables (yes/no) or (correct/incorrect) to explore the frequency of each answer option. Descriptive statistics were used to summarize demographic information and quantitative questions using frequencies and percentages. Qualitative data from the open-ended questions were analyzed using an inductive content analysis approach to explore both the content and frequency of codes [20,21]. This involved three main phases: preparation and making sense of the data, organizing the data (open coding, grouping, categorizing and abstraction) and reporting [21].

## 3. Results

**Figure 1:**
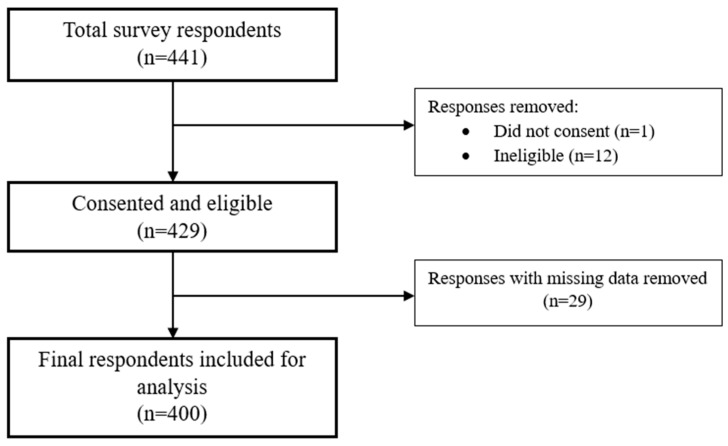
Flow chart of survey respondents.

A total of 441 people participated in the online survey across the three-month period, as shown in Figure 1. Overall, 13 respondents (9%) were excluded due to them not providing consent (*n* = 1) or due to non-eligibility (*n* = 12). Incomplete survey responses were then removed (*n* = 29), resulting in a total of 400 respondents being included in the final sample for analysis.

### 3.1. Participant Characteristics

Table 1 highlights the characteristics of the 400 Pacific respondents. The largest group were aged between 16 and 24 years (34%) and identified as female (66%). The highest level of education was most commonly secondary school qualifications (43.5%), followed by tertiary education degrees (21%), trades, apprenticeships and diplomas below a bachelor’s level (18.3%) and no educational qualification (17.3%). Tongan was the most common ethnicity (47.3%), followed by Samoan (28%), Cook Islands Māori (9.5%) and Niuean (8.3%).

### 3.2. Rheumatic Fever Content

#### 3.2.1. Sore Throats

Overall, 71% of Pacific survey respondents agreed that a child’s sore throat should always be checked by a health professional, compared with 20.8% who selected “sometimes”, 1.8% who selected “never” and 6.5% who said, “I don’t know” (Table 2). Open-ended responses to a follow-up question asking what could happen if a sore throat went untreated highlighted streptococcal infections or rheumatic fever in 25.8% of answers, with a further 4.3% discussing heart problems (Supplementary File S3). The largest proportion of responses (30.8%), however, referred to other diseases or non-specific health conditions such as coughing, tonsilitis, COVID-19 and a lack of appetite. Other responses mentioned descriptions of getting sicker (19%), general infections (11.3%) and 8% of answers said that sore throats would go away on their own (8%). The following are example quotes from responses:

*High possibility you could have strep throat, which leads to rheumatic fever, damaging the heart valves*

*(Female, Samoan/Tongan, 25–34 years).*

*It will result in more pain and when you go to the doctors it might be too late, resulting in surgeries and medications. But it’s too late. Go see a doctor as soon as you can to treat it in time*

*(Female, Tongan, 55–64 years).*

*If it is serious they may have to see the doctor but sometimes (a) sore throat will be healed on its own*

*(Female, Samoan, 25–34 years).*

*The tonsils will be infected causing a much bigger problem *

*(Male, Rotuman, 35–44 years).*

When antibiotics are provided by a health professional to a patient with a sore throat, 64.8% of Pacific survey respondents correctly selected that such medications should be taken until finished. Approximately 21% said that medication could be stopped when a person feels better, and fewer selected options with a set number of days (2.5%) or said they did not know (11.3%).

#### 3.2.2. Defining Rheumatic Fever

Of the 400 respondents in this survey, 65.3% (*n* = 261) identified that they had heard of rheumatic fever before. Of those who answered yes, 70.5% correctly identified that it is a disease that can develop when strep throat is not properly treated. The remaining 29.5% of responses (*n* = 77) were either partially or fully incorrect. Pacific people in this study also strongly agreed (43.7%) or agreed (36%) that rheumatic fever is preventable. Only 7.6% selected disagreeing responses.

#### 3.2.3. What Do You Know of Rheumatic Fever?

**Figure 2:**
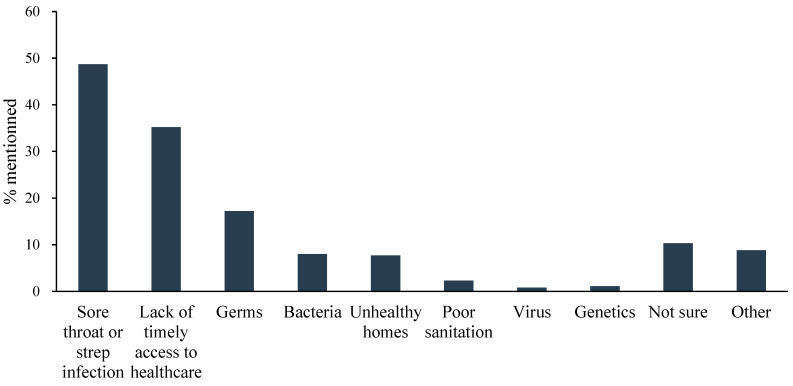
Risk factors for rheumatic fever.

The participants who had heard of rheumatic fever were asked through an open-ended question about the risk factors for how they think someone contracts rheumatic fever (Figure 2). The responses typically reiterated the pathway from a sore throat or strep throat to rheumatic fever (48.7%) or described a lack of timely access to treatment (35.2%). Primordial risk factors, such as unhealthy, damp or crowded houses, were mentioned only 7.7% of the time in answers. Other responses discussed the spreading of germs (17.2%) and the role of bacteria (8%), genetics (1.1%) and viruses (0.8%). 

Examples of responses include the following:

*Rheumatic fever begins from a sore throat, if ignored and not seek medical attention, it can lead to rheumatic fever.*

*(Female, Tokelauan/Tongan, 35–44 years).*

*Damp homes, overcrowded homes with no proper ventilation*

*(Female, Samoan, 25–34 years).*

*When the house is too cold you can get the bugs*

*(Female, Cook Islands Maori/Tongan, 75 and over).*

The signs and symptoms of having rheumatic fever were asked about in an open-ended question, with a fever being described in approximately half of the responses. While sore throats were again commonly mentioned (43.7%), other key symptoms of rheumatic fever appeared less, such as chest pain (15.3%), body aches (15.3%) and joint pain (14.2%).

When asked what could happen if rheumatic fever is not treated, more than half of the respondents identified heart-related problems (*n* = 147, 56.5%) in their answers. Sixty participants mentioned premature mortality (23.1%) and other answers included pain, getting worse or being hospitalized. A total of 7.3% said that they did not know what would happen. The following are examples of responses:

*It can hurt your heart*

*(Male, Tongan/New Zealand European, 16–24 years).*

*If a person does not receive treatment for rheumatic fever or experiences it multiple times, they could develop a long-term health condition that affects the heart. This condition is rheumatic heart disease*

*(Male, Tongan, 16–24 years).*

*Can develop a hole in your heart which can result in open heart surgery*

*(Female, Tuvaluan, 65–74 years).*

### 3.3. Accessing Health Information

Pacific survey respondents were asked where they had seen or heard about rheumatic fever before, with “my doctor or nurse” being the most selected option (54.4%), followed by family and friends (47.1%). Social media was selected by participants (26.1%) less commonly than traditional media avenues (36.8%), such as newspapers, magazines, radio and television. When respondents were asked to select where, in general, they obtain health-related information from, a doctor was the most common (70%), followed by social media (37.3%) and family members (36.8%). In contrast, an open-ended question regarding where they believe other Pacific people most commonly access health information, the largest proportion of responses described social media or the internet (36.1%). While the potential for misinformation was highlighted, respondents discussed online platforms as being an easy, inexpensive and comfortable way of learning about health and connecting with real stories.

*Most people in our Māori and Pasifika (communities) use social media as their main source of obtaining health information, because social media is popular within younger as well as older generations. Although it has its positives/highlights, it also leaves an opportunity for widespread misinformation, specifically regarding health, as there are many conspiracy theories in society*

*(Female, Samoan, 16–24 years).*

*Facebook, Instagram and TikTok, because these sites are famous for people of all backgrounds to share their medical journey and experiences about health”*

*(Female, Tongan, 16–24 years).*

*Some Pacific people may be too shy to ask information so they would go out of their way to search for it themselves”*

*(Male, Cook Islands Māori/Tongan/New Zealand European, 35–44 years).*

Health professionals were mentioned by 31.6% of respondents regarding where they thought other Pacific people access information, with long-standing relationships with family doctors described as trusted sources of information. Despite this, expensive costs, language differences and difficulties obtaining timely appointments were discussed as barriers to access. Pacific cultural factors also played a role, with some responses referring to feeling fakamā (shy or shameful) or scared of potential judgment from health professionals.

*The doctors have the best and most trusted health information but most commonly in Pacific families, we are shy to discuss with health professionals, so we settle for searching for health information on our own*

*(Female, Cook Islands Māori/Tongan/New Zealand European, 25–34 years).*

*Family doctors (because) they know a lot about the health of a person*

*(Male, Tongan, 16–24 years).*

*Going to the doctors is costly, sometimes unnecessary, and doctors tend to Google things in front of you anyway*

*(Female, Samoan, 25–34 years).*

*Because of the language barrier and cultural circumstances, i.e., being a female Pacific Islander and having a male doctor, (that) would stop some people from going to the doctor”*

*(Samoan/Tahitian, 25–34 years).*

Word of mouth from family, friends and the wider community were discussed in 28.8% of responses, with the collectivist, face-to-face nature of Pacific cultures being referenced. Information was perceived to be shared through communal gatherings, churches and via traditional medicine approaches passed down through generations.

*Word of mouth in the communities. We rely on our communities for knowledge.*

*(Female, Tongan, 25–34 years).*

*Most people I know don’t go to the doctors until their symptoms are really bad, so they just ask their family for advice *

*(Female, Maori/Samoan, 25–34 years).*

*Pacific people like to catch up with people and have a chat. The most access they have to health information is through conversations.*

*(Male, Tongan/New Zealand European, 35–44 years).*

*Pacific people share their experiences about their own health journeys, sometimes this is a good thing and sometimes it’s not good, as at times the wrong information could be shared. In the Pacific way, talanoa (talking) is the main communication*

*(Female, Samoan/Tongan, 25–34 years).*

## 4. Discussion

Public understanding of rheumatic fever is an important component of multifaceted strategies to prevent recurrences and disease progression. Although Pacific people are disproportionately affected by rheumatic fever in NZ, there is a lack of data around the understanding of both the disease and supportive services. A Pacific community group and health professional network aimed to investigate the awareness and understanding of rheumatic fever among Pacific people living in Auckland, NZ, and to explore how people access health-related information. Over 16 different ethnic groups were represented in this survey, with the majority of respondents female (66%) and below the age of 45 years (79.8%).

This survey found that 71% of Pacific respondents agreed that a child’s sore throat should always be checked by a health professional, and 64.8% correctly indicated that antibiotics prescribed by a health professional should be taken until finished. Such agreement is lower than rates reported over a decade ago in NZ, which assessed the rheumatic fever knowledge of parents as part of an evaluation of a four-month national mass media awareness campaign in 2014 [22]. Telephone interviews with 800 parents of 4–18-year-olds were conducted, with 88% of Pacific people surveyed (*n* = 82) agreeing that all sore throats in children need to be checked by a doctor or nurse straight away [22]. Although our survey captured a larger sample and broader range of Pacific people, the decrease in attitudes over time perhaps highlights the lack of current awareness strategies and educational approaches available. Sustained and ongoing health promotion campaigns can support better the awareness of rheumatic fever, but should be designed alongside Pacific communities and avoid further victim blaming and stigmatization [12]. Discussion questions later in the survey also highlighted structural barriers faced by Pacific people, such as cost, location and cultural factors, that influence access to health services and should be addressed alongside awareness to improve timely healthcare.

Approximately two thirds of Pacific participants in this study reported that they had heard of rheumatic fever before. Most described a sore throat or streptococcal infection in open-ended answers regarding how people contract rheumatic fever, with less recognition of primordial risk factors. In other countries with high burdens of rheumatic fever, similar levels of awareness are reported. Sayed and colleagues conducted a cross-sectional survey of 6958 people in hospitals across Egypt, finding that 62% had heard about rheumatic heart disease and 34% were aware of the link between sore throats and heart disease [23]. In Saudi Arabia, an online survey of 761 people aged 18 years and above found that 53.5% had heard of rheumatic heart disease [24]. Despite similarities with other countries, the awareness of rheumatic fever among Pacific respondents in this study is considerably lower compared with previous surveys in the NZ context [22]. Approaches are needed to increase the understanding of rheumatic fever in general, and to focus on the gaps highlighted in the survey responses, such as primordial risk factors like sub-standard housing and socio-economic factors.

A fever was the most commonly identified sign or symptom identified in open-ended discussion responses (50.3%) followed by a sore throat (43.7%). Symptoms of joint pain, skin rashes and jerky movements, which are key components of the Jones criteria used to diagnose rheumatic fever, were seldom mentioned in responses. A study by Ray and colleagues in India similarly noted that schoolchildren aged 10–16 years most commonly identified fever as a symptom of rheumatic fever, followed by easy tiredness and sore throat [6]. However, conversely, a study in Saudi Arabia showed that 80% of the 1364 participants aged 18 and over were aware that joint pain, inflammation of the heart, rash and involuntary movement disorder were symptoms of rheumatic fever [25]. The low frequency of these key symptoms being identified in Pacific survey responses links with previous qualitative research by our team [15]. In co-design workshops, Pacific community members discussed the emphasis on sore throats in past health promotion campaigns, which in their view may have unintentionally excluded knowledge of other important symptoms [15]. This is particularly important when considering that as many as a third of acute rheumatic fever patients do not remember having an antecedent sore throat [26]. The early detection of disease symptoms is important for enabling timely diagnosis and treatment, thus it is critical that health literacy efforts support a more comprehensive understanding of the varied symptoms.

While most Pacific respondents identified doctors as their main source of rheumatic fever and general health information, broader discussions of how they believe other Pacific people access information revealed a tendency towards social media and online methods. Participants highlighted the ease of access and discussed that it helped to address barriers to health services, such as expensive costs, long waiting times and cultural differences. A qualitative study in Hong Kong exploring perceptions of health information seeking among 49 participants aged 18 years and above similarly found that the majority (97%) identified the internet as the first means they used to seek health information [27]. The reasons provided by participants included increased convenience and the ability to explore health topics in-depth, beyond what can typically be covered in a short appointment with a health professional [27]. Social media is increasingly becoming a medium for health promotion and education, due to its engaging nature and popular use among young people. It highlights an opportunity for the dissemination of rheumatic fever messages for Pacific people, in ways that circumvent traditional barriers to health services.

The findings from this research have clear implications for health promotion and educational strategies moving forward. While the awareness of rheumatic fever and long-term impacts match those of other countries with high burdens, it is much lower compared with research undertaken in the NZ context over a decade ago. Key gaps were also clear in open-ended responses, with symptoms such as joint pain, chest pain and shortness of breath being mentioned less frequently than sore throats. An opportunity for social media and internet-based approaches alongside wider community approaches is clear, to align with the ways in which Pacific communities currently access health information. The findings from this study will directly inform a Pacific community-designed social media intervention for rheumatic fever in NZ that is currently being co-developed by PPHAG and PPBRN. Such education strategies need to be undertaken alongside broader systemic changes, as the survey findings continue to highlight barriers to health services. Prevention-related strategies must not exclude primordial prevention factors that address underlying social determinants such as sub-standard housing, poor health literacy and difficulties in access to healthcare, and the public understanding of these factors should be improved to reduce the potential for individual blame. Future research may undertake similar participatory research approaches in other settings highly affected by rheumatic fever, such as low- and middle-income countries.

A strength of this study was the community-led design, whereby Pacific community and health professional groups established the underlying project and contributed to designing and refining the questionnaire. This ensured that the survey tool was tailored specifically to Pacific communities regarding its content, presentation and format. The online-based approach was also a strength, being an easy, low-cost method of reaching a wide range of people. The research team identified that this method may limit external validity, with bias for participation potentially leaning towards younger, more educated groups and those who feel most comfortable with digital technology. To pre-emptively address this, our recruitment strategy included reaching out to wider Pacific networks of the research team, including community groups, churches, schools and sports clubs, to ensure a variety of ages and education levels were invited to take part. Although education levels in this survey align with national levels for Pacific people, the broader generalizability of the results may be limited by the non-probabilistic sampling method and high proportion of female responses (66%) in the final sample. Although higher female participation is somewhat typical in online survey formats [28], research into rheumatic fever awareness may endeavor to address this sampling bias in future and aim for more balanced gender representation.

## 5. Conclusions

Using a co-designed survey tool, this cross-sectional study explored Pacific people’s awareness and understanding of rheumatic fever and identified where they typically access health information. The findings emphasized key opportunities for health education and promotion strategies moving forward, including the general awareness of rheumatic fever, an increased focus on the varied symptoms and supporting the understanding of treatment pathways to enable both primary and secondary prevention. Equipping communities with rheumatic fever knowledge is critical to enable timely healthcare and increase the uptake of and adherence to treatment. Future research will utilize the findings from this study in the development of community-led social media strategies for Pacific people living in NZ.

## Figures and Tables

**Table 1:** Characteristics of participants.

Variable	Attribute	*n*	%
Gender	Male	134	33.5
	Female	264	66
	Gender diverse	0	0
	Prefer not to say	2	0.5
Age range	16–24 years	136	34
	25–34 years	105	26.3
	35–44 years	78	19.5
	45–54 years	30	7.5
	55–64 years	26	6.5
	65–74 years	18	4.5
	75 and over	7	1.8
Education level	No educational qualification	69	17.3
	High school qualification	174	43.5
	Trade certificate, apprenticeship or diploma below a Bachelor’s	73	18.3
	Tertiary education	84	21
Ethnicity *	Tongan	189	47.3
	Samoan	112	28
	Cook Islands Māori	38	9.5
	Niuean	33	8.3
	Fijian	31	7.8
	Tokelauan	11	2.8
	I-Kiribati	6	1.5
	Rotuman	4	1.0
	French Polynesian	1	0.3
	Nauruan	1	0.3
	Papua New Guinean	1	0.3
	Pitcairn Islander	1	0.3
	Māori	30	9.5
	New Zealand European	26	6.5
	Chinese	1	0.3
	German	1	0.3
	Prefer not to say	2	0.5
Parent or caregiver	Yes	142	35.5
	No	258	64.5
Knows someone with rheumatic fever	Yes	140	35
	No	260	65

* Multiple ethnicities could be selected.

**Table 2:** Selected rheumatic fever questions.

Question	*n*	%
When should a child’s sore throat be checked by a health professional?		
Always	284	71
Sometimes	83	20.8
Never	7	1.8
I don’t know	26	6.5
If antibiotics are given for a sore throat, when should someone stop taking them?		
After 3 days	4	1
After 5 days	6	1.5
When they feel better	85	21.3
Until the medication is finished	259	64.8
I don’t know	46	11.5
Have you heard of rheumatic fever?		
Yes	261	65.3
No	139	34.8
What is rheumatic fever?		
Correct answer selected (A disease that can develop when strep throat isn’t properly treated)	184	70.5
Incorrect answer selected	77	29.5
How much do you agree or disagree with the statement *“Rheumatic fever is preventable”*?		
Strongly disagree	11	4.2
Disagree	9	3.4
Neutral	33	12.6
Agree	94	36
Strongly agree	114	43.7

## Data Availability

The data presented in this study are not publicly available due to ethical considerations but may be available on request from the corresponding author.

## Supplementary Materials

The following supporting information can be downloaded at: https://www.mdpi.com/article/10.3390/healthcare13222924/s1, S1: Strobe Checklist; S2: Questionnaire tool; S3: Tables.

## Abbreviations

The following abbreviations are used in this manuscript:

PPBRN	Pacific Practice-based Research Network
PPHAG	Pacific People’s Health Advisory Group
NZ	Aotearoa New Zealand
STROBE	The Strengthening the Reporting of Observational Studies in Epidemiology Statement

## References

[1] Chowdhury M.S., Koziatek C.A., Rajnik M. (2023). Acute Rheumatic Fever. StatPearls.

[2] World Health Organization (2024). WHO Guideline on the Prevention and Diagnosis of Rheumatic Fever and Rheumatic Heart Disease.

[3] Baker M.G., Gurney J., Moreland N.J., Bennett J., Oliver J., Williamson D.A., Pierse N., Wilson N., Merriman T.R., Percival T. (2022). Risk factors for acute rheumatic fever: A case-control study. Lancet Reg. Health–West. Pac..

[4] Haghaninejad H., Ardian N., Mazloomy Mahmoudabad S.S., Sefidkar R., Jokar M. (2024). Effect of Educational Intervention on Awareness, Attitude, and Practices of Mothers Regarding the Prevention of Rheumatic Fever: A Quasi-Experimental Study. Inq. J. Health Care Organ. Provis. Financ..

[5] Kayima J., Kaddumukasa M. (2024). An urgent need for early diagnosis and treatment of acute rheumatic fever. Lancet Glob. Health.

[6] Ray M., Guha S., Ray M., Karak A., Choudhury B., Ray B., Hazra P.C., Selker H.P., Goldberg R.J., Bhatt D.L. (2020). A questionnaire survey for improving awareness of rheumatic heart disease among school-aged children in India. Indian Heart J..

[7] Allen L.B., Allen M., Lesa R.F.a., Richardson G.E., Eggett D.L. (2011). Rheumatic fever in Samoa: Education as prevention. Pac. Health Dialog.

[8] Nkoke C., Luchuo E.B., Jingi A.M., Makoge C., Hamadou B., Dzudie A. (2018). Rheumatic heart disease awareness in the South West region of Cameroon: A hospital based survey in a Sub-Saharan African setting. PLoS ONE.

[9] Te Whatu Ora Health New Zealand (2025). Reducing Rheumatic Fever Wellington. https://www.tewhatuora.govt.nz/for-health-professionals/clinical-guidance/diseases-and-conditions/rheumatic-fever-guidance/reducing-rheumatic-fever.

[10] Te Whatu Ora Health New Zealand (2023). Rheumatic Fever Roadmap: A Roadmap for the Prevention and Management of Rheumatic Fever and Rheumatic Heart Disease from 2023–2028.

[11] Gurney J.K., Chong A., Culliford-Semmens N., Tilton E., Wilson N.J., Sarfati D. (2017). High levels of rheumatic fever and sore throat awareness among a highrisk population screened for rheumatic heart disease. N. Z. Med. J..

[12] Anderson A., Spray J. (2020). Beyond awareness: Towards a critically conscious health promotion for rheumatic fever in Aotearoa, New Zealand. Soc. Sci. Med..

[13] Naea N., Dobson A., Leversha A., Williams S., Knott K., Clayton-Bray L., Dickinson A. (2016). Awareness and understanding of rheumatic fever among Pacific people in Auckland. Neonatal Paediatr. Child Health Nurs..

[14] Tu’akoi S., Ofanoa M., Ofanoa S., Lutui H., Heather M., Jansen R.M., van der Werf B., Goodyear-Smith F. (2022). Co-designing an intervention to prevent rheumatic fever in Pacific People in South Auckland: A study protocol. Int. J. Equity Health.

[15] Tu’akoi S., Ofanoa M., Ofanoa S., Lutui H., Heather M., Goodyear-Smith F. (2025). ‘Not a short fix’: A participatory approach to exploring challenges and opportunities for rheumatic fever prevention with Pacific people in South Auckland, New Zealand. J. Health Equity.

[16] Lee C.-C., Li D., Arai S., Puntillo K. (2009). Ensuring cross-cultural equivalence in translation of research consents and clinical documents: A systematic process for translating English to Chinese. J. Transcult. Nurs..

[17] Scott B. (2024). Readability Scoring System: Readability Formulas. https://readabilityformulas.com/readability-scoring-system.php.

[18] Ministry of Social Development (2025). Improving How We Report Ethnicity.

[19] Mirzaei A., Carter S.R., Patanwala A.E., Schneider C.R. (2022). Missing data in surveys: Key concepts, approaches, and applications. Res. Soc. Adm. Pharm..

[20] Galura S.J., Horan K.A., Parchment J., Penoyer D., Schlotzhauer A., Dye K., Hill E. (2022). Frame of reference training for content analysis with structured teams (FORT-CAST): A framework for content analysis of open-ended survey questions using multidisciplinary coders. Res. Nurs. Health.

[21] Elo S., Kyngäs H. (2008). The qualitative content analysis process. J. Adv. Nurs..

[22] TNS New Zealand Limited (2015). 2014 Rheumatic Fever Campaign Evaluation: Report Commissioned by the Health Promotion Agency.

[23] Sayed A.K., Se’eda H., Eltewacy N.K., El Sherif L.a., Ghalioub H.S., Sayed A., Afifi A.M., Almoallim H.S., Alharbi S.A., Abushouk A.I. (2021). Awareness of rheumatic heart disease in Egypt: A national multicenter study. J. Cardiovasc. Dev. Dis..

[24] Mougrabi M.M., Aljuaid R.S., Alrabie A.D., Althumali N.K., Alkhaldi L.H., Alotaibi W.D. (2021). Awareness of rheumatic fever and rheumatic heart disease among the population in Taif, Saudi Arabia 2020. J. Fam. Med. Prim. Care.

[25] Fakieha A.Y., Zafer D.O., Alkalash S.H., Fudah A.A., Mujlid R.M., Fakiha M.Y., Khafajy A., Shatla M.M., Fakieha A., Zafer D. (2024). Knowledge and Attitude of Rheumatic Fever and Rheumatic Heart Disease Among the Makkah City Population, Saudi Arabia. Cureus.

[26] Stollerman G.H. (2001). Rheumatic fever in the 21st century. Clin. Infect. Dis..

[27] Chu J.T., Wang M.P., Shen C., Viswanath K., Lam T.H., Chan S.S.C. (2017). How, when and why people seek health information online: Qualitative study in Hong Kong. Interact. J. Med. Res..

[28] Wu M.-J., Zhao K., Fils-Aime F. (2022). Response rates of online surveys in published research: A meta-analysis. Comput. Hum. Behav. Rep..

