# Supplementary material for: “It Can Hurt Your Heart”: A Co-Designed Cross-Sectional Survey Exploring Pacific People’s Understanding of Rheumatic Fever in Auckland, New Zealand
Source: Healthcare (Basel). 2025 Nov 15;13(22):2924. doi: 10.3390/healthcare13222924 (PMC12652848; doi:10.3390/healthcare13222924)
Supplement: Supplementary file 1 [file healthcare-13-02924-s001.zip › Supplementary 2 - Questionnaire tool.pdf]

Supplementary S2: Questionnaire tool

Consent question

Do you consent to participate in this survey?

- ◯ Yes
- ◯ No

Skip To: End of Survey If Do you consent to participate in this survey? = No

Eligibility questions

Do you identify with at least one Pacific ethnicity?

- ◯ Yes
- ◯ No

Skip To: End of Survey If Do you identify with at least one Pacific ethnicity? = No

Do you live in Auckland?

- ◯ Yes
- ◯ No

Skip To: End of Survey If Do you live in Auckland? = No

What age range do you fit within?

- ◯ Under 16 years
- ◯ 16-24 years
- ◯ 25-34 years
- ◯ 35-44 years
- ◯ 45-54 years
- ◯ 55-64 years
- ◯ 65-74 years
- ◯ 75 and over

Awareness & understanding of rheumatic fever questions

Q1. When should a child's sore throat be checked by a health professional?

- ◯ Always
- ◯ Sometimes
- ◯ Never
- ◯ I don't know

Q2. What could happen if a sore throat is not treated?

____

Q3. If antibiotics are given for a sore throat, when should someone stop taking them?

- ◯ After 3 days
- ◯ After 5 days
- ◯ When they feel better
- ◯ Until the medication is finished
- ◯ I don't know

Q4. Have you heard of rheumatic fever?

- ◯ Yes
- ◯ No

Skip To: End of Block If Q4. Have you heard of rheumatic fever? = No

Q5 What is rheumatic fever?

- [ ] It is a type of flu
- [ ] It causes rheumatoid arthritis
- [ ] A disease that can develop when strep throat isn't properly treated
- [ ] It mostly affects adults

Q6. Where have you seen or heard about rheumatic fever before?

- [ ] From family and friends
- [ ] From my doctor or nurse
- [ ] From the media such as newspapers, magazines, radio and TV
- [ ] From social media
- [ ] From billboards and other advertisements
- [ ] Other (please state) ______________________________

Q7. How much do you agree or disagree with this statement?
"Rheumatic fever is preventable"

- ○ Strongly Disagree
- ○ Disagree
- ○ Neutral
- ○ Agree
- ○ Strongly Agree

Q8. How do you think you get rheumatic fever?

____

Q9. What are signs of rheumatic fever?

____

Q10. What could happen if rheumatic fever is not treated?

____

Accessing health information questions

Q11. Where do YOU get health-related information from? Select all that apply.

- ☐ Social media
- ☐ Internet search engines
- ☐ Television
- ☐ Radio
- ☐ Family members
- ☐ A doctor
- ☐ Other health professionals (nurses, pharmacists)
- ☐ Word of mouth
- ☐ Other ___________

Q12. Where do you think OTHER Pacific people most commonly access health information from? Why?

____

Demographic questions

Q13. What gender do you identify with?

- ◯ Male
- ◯ Female
- ◯ Non-binary / Gender diverse
- ◯ Prefer not to say
- ◯ Other ________________________________

Q14. Which ethnic group do you belong to? Select all that apply.

☐	Cook Islands Māori	☐	Samoan
☐	Fijian	☐	Solomon Islander
☐	Hawaiian	☐	Tahitian
☐	Kiribati	☐	Tokelauan
☐	Māori	☐	Tongan
☐	Nauruan	☐	Tuvaluan
☐	Niuean	☐	Ni Vanuatu
☐	Papua New Guinean	☐	New Zealand European
☐	Pitcairn Islander	☐	Other: ____________
☐	Rotuman	☐	Prefer not to say

Q15. What is your highest COMPLETED level of education?

- ◯ No educational qualification
- ◯ NCEA Level 1 Certificate/School Certificate
- ◯ NCEA Level 2 Certificate/Sixth Form Certificate
- ◯ NCEA Level 3 or 4 Certificate/University Bursary
- ◯ Trade certificate or apprenticeship
- ◯ Diploma below a Bachelor's
- ◯ Bachelor's degree
- ◯ Postgraduate diploma
- ◯ Master's degree
- ◯ PhD or doctoral degree
- ◯ Other: ____________

Q16. What is your occupation?

____

Q17. Are you a parent or caregiver?

- ◯ Yes
- ◯ No

Q18. If yes, how many children do you have/are responsible for and what are their ages? _____

Q19. Do you, a family member or someone you know have rheumatic fever? Select all that apply

- ☐ Myself
- ☐ My child(ren)
- ☐ An immediate family member
- ☐ An extended family member
- ☐ A friend or someone else you know
- ☐ Other: ________________
- ☐ No one I know has rheumatic fever
