# Supplementary material for: “It Can Hurt Your Heart”: A Co-Designed Cross-Sectional Survey Exploring Pacific People’s Understanding of Rheumatic Fever in Auckland, New Zealand
Source: Healthcare (Basel). 2025 Nov 15;13(22):2924. doi: 10.3390/healthcare13222924 (PMC12652848; doi:10.3390/healthcare13222924)
Supplement: Supplementary file 1 [file healthcare-13-02924-s001.zip › Supplementary 3 - Tables.pdf]

Supplementary S3: Table of coded responses

Table S1: Frequency counts of multiple choice and coded open-ended questions

**Question and responses**	n	%
What could happen if a sore throat is not treated? *		
Mentions streptococcus or rheumatic fever	103	25.8
Heart problems	17	4.3
Get sicker	76	19
Severe outcomes (hospitalisation, premature mortality)	33	8.3
Infection	45	11.3
Other health conditions	123	30.8
Nothing	13	3.3
Don't know	32	8
Other	14	3.5
Where have you seen or heard about rheumatic fever before?		
My doctor or nurse	142	54.4
Family and friends	123	47.1
The media (newspapers, magazines, radio, TV)	96	36.8
Social media	68	26.1
Billboards and other ads	22	8.4
Other	23	8.8
How do you think you get rheumatic fever? *		
Sore throat/strep infection	127	48.7
Unhealthy housing	20	7.7
Bacteria	21	8
Lack of timely treatment	92	35.2
Germs	45	17.2
Poor sanitation/conditions	6	2.3
Virus	2	0.8
Genetics	3	1.1
Other	23	8.8
Not sure	27	6.8
Improperly answered	2	0.8
What are signs of rheumatic fever? *		
Sore throat	114	43.7
Fever	131	50.2
Joint pain	37	14.2
Shortness of breath	19	7.3
Body aches	40	15.3
Fatigue/lack of energy	18	6.9
Skin infection or rash	13	5
Jerky movements	1	0.4
Heart problems	17	6.5
Coughing	22	8.4
Chest pain	40	15.3
Other	43	16.5
I don't know	13	5
What could happen if rheumatic fever is not treated? *		
Heart disease/heart problems	147	56.5

Premature mortality	60	23.1
Other	50	19.2
I don't know	19	7.3
Where do you get health-related information from?		
A doctor	277	69.3
Social media	149	37.3
Family	147	36.8
Other health professionals (nurses, pharmacists)	141	35.3
Internet search engines	138	34.5
Word of mouth	87	21.8
Radio	62	15.5
Television	48	12
Other	17	4.3

*Refers to open-ended questions that were coded using content analysis and frequency counts
